# Prophylactic hyperthermic intraperitoneal chemotherapy (HIPEC) for the prevention and control of peritoneal metastasis in patients with gastrointestinal malignancies: a systematic review of randomized controlled trials

**DOI:** 10.17179/excli2021-4108

**Published:** 2021-08-19

**Authors:** Jerry Lorren Dominic, Amudhan Kannan, Anjli Tara, Abdul Rub Hakim Mohammed, Myat Win, Arseni Khorochkov, Waleed Sultan, Asma Ahmed, Ketan Kantamaneni, Michael W. Syzmanski, Rajbir Singh, Raul A. Marquez, Armand Asarian, Pragatheeshwar Thirunavukarasu, George Keckeisen

**Affiliations:** 1 General Surgery, Stony Brook Medicine/Southampton Hospital, Southampton, New York, United States; 2 Jawaharlal Institute of Postgraduate Medical Education and Research (JIPMER), Puducherry, India; 3 General Surgery, California Institute of Behavioral Neurosciences and Psychology, Fairfield, California, United States; 4 Emergency Medicine, The George Washington University - Kokilaben Dhirubhai Ambani Hospital & Medical Research Institute, Mumbai, India; 5 General Surgery, Nottingham University Hospitals NHS Trust, Nottingham, United Kingdom; 6 General Surgery, Halifax Health Medical Center, Daytona Beach, Florida, United States; 7 General Surgery, University of Missouri - Kansas City, Missouri, United States; 8 General Surgery, Dr. Pinnamaneni Siddhartha Institute of Medical Sciences & Research Foundation, Gannavaram, Andhra Pradesh, India; 9 Orthopedic Surgery, Cornerstone Regional Hospital/South Texas Health System, Edinburg, Texas, United States; 10 General Surgery, The Brooklyn Hospital Center, Brooklyn, New York, United States; 11 Director of Surgical Oncology, Cape Fear Valley Medical Center, Fayetteville, North Carolina, United States and Adjunct Clinical Assistant Professor of Surgery, Oklahoma State University Center for Health Sciences, Tulsa, Oklahoma, United States

**Keywords:** hyperthermic intraperitoneal chemotherapy, peritoneal neoplasms, gastrointestinal neoplasm, gastric neoplasm, colorectal neoplasm, mortality

## Abstract

Peritoneal metastasis is associated with poor prognosis, with studies in the literature reporting the survival of peritoneal metastasis without treatment to be three to six months. Hyperthermic intraperitoneal chemotherapy (HIPEC) has shown positive outcomes by improving the prognosis in patients with gastrointestinal malignancies. This systematic review of randomized controlled trials was done to determine the prophylactic role of hyperthermic intraperitoneal chemotherapy in preventing and controlling peritoneal metastasis gastrointestinal origin. Randomized controlled trials published between January 2019 to June 2021 were included. The databases used were MEDLINE (PubMed), EMBASE (Ovid), and the Cochrane library. Cochrane handbook for systematic review of intervention was used to assess the risk of bias in included trials. The results were reported using the Preferred Reporting Items for Systematic Reviews and Meta-Analysis (PRISMA) guidelines. A total of five trials met the inclusion criteria. Two studies were on patients with gastric cancer, and the other three studies were on patients with colorectal cancer. HIPEC was given to a total of 116 gastric cancer patients and 308 colorectal cancer patients. In all the included studies on patients with gastric cancer, the peritoneal recurrence-free survival was significantly higher in the group that received HIPEC. There was no significant improvement in peritoneal-free survival in patients with colorectal cancer who received HIPEC. HIPEC appears to be effective in preventing peritoneal metastasis in patients with locally advanced gastric cancer without minimal postoperative complications. However, in patients with advanced colorectal malignancy, HIPEC does not seem to play a crucial role in preventing and controlling peritoneal metastasis.

## Introduction

Peritoneal metastatic disease is a condition of the peritoneum characterized by multiple foci of metastatic tumors, usually of gastrointestinal or gynecological origin. It can occur through various mechanisms. These include implantation of the cancer cells onto the peritoneum with the help of adhesion molecules, direct extension of the tumor cells into the peritoneum through the serosa, through lymphatic or hematogenous spread (Sugarbaker, 1998[30]; Glehen et al., 2004[10]; Iitsuka et al. 1979[12]). Peritoneal metastasis is associated with poor prognosis, with studies in the literature reporting the survival of peritoneal metastasis without treatment to be three to six months (Glehen et al., 2004[10]). Of the tumors metastasizing to the peritoneum, abdominal organ malignancies represent the most frequently involved primary organ sites. These malignancies include gastric cancer, appendiceal tumors, and colorectal cancer. Among the gynecological malignancies, ovarian cancers are the most commonly associated with peritoneal metastasis. The two most common gastrointestinal cancers that often metastasize to the peritoneum are gastric cancer and colorectal cancer. Gastric cancer is the fifth most common cancer worldwide, and it is the third most common cause of cancer-related deaths worldwide. It is very prevalent in Asian countries, especially in China and eastern Asian countries (Ferlay et al., 2015[7]). It has an almost 15 % peritoneal recurrence rate even after surgery in advanced stages characterized by serosal invasion (Brigand et al., 2004[4]). Pecqueux et al. (2015[21]), in their meta-analysis, had reported that the presence of free intraperitoneal tumor cells in patients with gastric cancer is associated with high recurrence rates and poor survival outcomes. Another most important malignancy of the gastrointestinal tract is colorectal cancer. According to the data, it is the second most common cancer in women and the third most common cancer in men (Pecqueux et al., 2015[21]). Almost 55 % of the cases occur in more developed regions. 

The peritoneum is a common site of metastasis. The factors that increase the risk of peritoneal metastasis include advanced tumor with perforation (T4 stage), right-sided tumors, radical dissection of the tumor (Segelman et al., 2012[28]; Klaver et al., 2018[13]). The incidence of peritoneal metastasis reported in the literature is around 8 % (Segelman et al., 2012[28]). Considering the fact that imaging is not very sensitive in helping in diagnosing peritoneal metastasis, questions arise on finding the exact incidence of peritoneal metastasis in colorectal cancer. With difficulty in imaging, peritoneal metastasis is often detected very late in the course of the disease, and it is associated with a poor prognosis (Franko et al., 2016[9]). Decades back, there were only a few options available for the treatment of peritoneal metastasis or neoplasms. This includes systemic chemotherapy and palliation. Now, the treatment of peritoneal metastasis has undergone a drastic change with many advancements. Unresectable peritoneal metastasis is resistant to systemic chemotherapy, and thus without surgery, the survival benefit of systemic chemotherapy is not much (Franko et al., 2012[8]). However, when resection of the peritoneal metastasis and primary tumor is possible, the patients can be offered surgical resection of the metastasis along with hyperthermic intraperitoneal chemotherapy (HIPEC). The first published study on human subjects to talk about hyperthermic intraperitoneal chemotherapy was by Spratt et al. (1980[29]), published in the year 1980. The procedure with cytoreduction was done on a 35-year-old male for pseudomyxoma peritoni. After this, Dr. Paul Sugarbaker headed a further investigation into HIPEC for gastrointestinal malignancies with peritoneal metastasis with reports of survival benefits, and in 1995, he described the technique of peritonectomy (Neuwirth et al., 2016[19]). Subsequently, the surgery per se has undergone many progressive moldings and refinements to the current state. HIPEC involves a multidisciplinary approach. 

**Figure 1:**
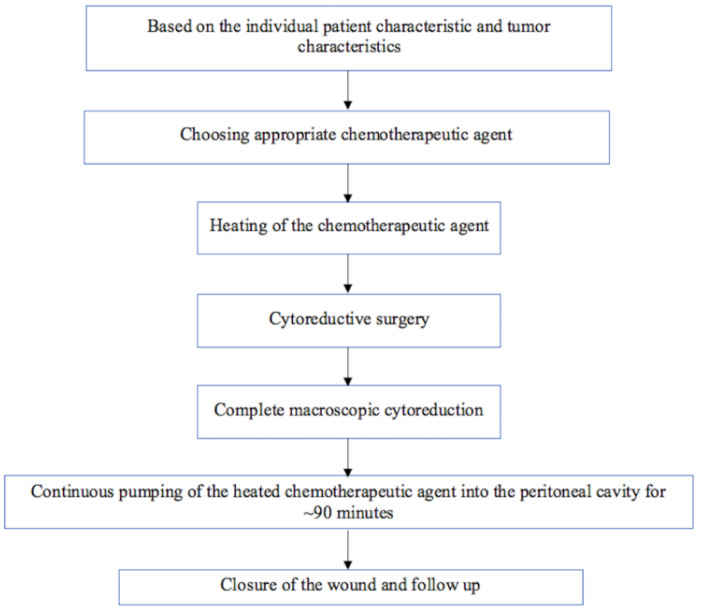
HIPEC procedure in brief

The procedure, in brief, is presented in Figure 1(Fig. 1). HIPEC involves the administration of heated chemotherapeutic agents into the peritoneal cavity. Heating the chemotherapeutic agent intensifies the penetration of agents into the cancer cells. It also boosts the uptake of the agents by the cancer cells, thus resulting in specific destruction of the cancer cells with minimal systemic adverse effects. Heat also induces apoptosis and causes denaturation of intracellular proteins (van Driel et al., 2018[32]). As metastatic involvement of the peritoneum is associated with poor prognosis, preventing the seeding and invasion of the peritoneum by the tumor may help in the improvement of the overall survival. 

Kyang et al. (2019[15]) had studied patients who underwent CRS (Cytoreductive Surgery), with or without HIPEC in Australia. All the maintained data of patients were analyzed retrospectively. After analysis, this study concluded that CRS with HIPEC could provide long‐term survival benefits to patients with peritoneal neoplasms of gastrointestinal and ovarian origin. Like Kyang et al.'s (2019[15]) study, various other prospective, retrospective, and non-randomized studies had reported optimistic results in favor of HIPEC to treat peritoneal metastasis of gastrointestinal tumor origin. However, only limited studies are found in the medical literature that was done to assess the prophylactic role of HIPEC in the prevention and control of peritoneal metastasis of gastrointestinal origin. Xie et al. (2020[34]) had done a non-RCT in China to study the role of prophylactic HIPEC after radical gastrectomy in patients with locally advanced gastric cancer. With results showing peritoneal recurrence rates of 4 % in the HIPEC group, the authors concluded that prophylactic HIPEC is beneficial in reducing the peritoneal recurrence rate and improving overall survival in patients with locally advanced gastric cancer.

Though there are various studies published in the literature, including reviews about the efficacy, safety, and outcomes of HIPEC, there are no systematic reviews of the randomized controlled trials published in the literature that evaluated the effectiveness of HIPEC in the prevention and control of peritoneal metastasis of gastrointestinal origin to the best of our knowledge. Therefore, this systematic review of randomized controlled trials was done to determine whether prophylactic HIPEC can prevent or reduce peritoneal metastasis in patients with gastrointestinal cancers.

## Methods

This systematic review was designed, and its results were reported using the Preferred Reporting Items for Systematic Reviews and Meta-Analysis (PRISMA) guidelines (Page et al., 2021[20]).

### Inclusion and exclusion criteria 

This study included only the randomized controlled trials comparing the outcomes of HIPEC and other methods for peritoneal metastasis and primary peritoneal tumors. All the different types of studies were excluded. This study did not have any constraint on the age of patients. This systematic review restricted the studies published between January 2018 to June 2021. Among the studies chosen, it was ensured that all the studies were done on human subjects and published in English language. Studies without full-text availability online were excluded. We also excluded the studies with less than four out of seven scores in Cochrane bias assessment for randomized clinical trials. Studies comparing the outcomes of HIPEC for patients with peritoneal sarcomas were excluded. 

### Information sources and search strategy 

The databases used were MEDLINE (PubMed), EMBASE (Ovid), and the Cochrane library. These databases were exhaustively searched for published studies from January 1, 2019, through June 1, 2021. Suitable keywords and medical subject heading (MeSH) terms were used in PubMed to fetch all pertinent articles that studied the outcomes of hyperthermic intraperitoneal chemotherapy (HIPEC) in patients with peritoneal metastasis and primary peritoneal tumors. The keywords used include "HIPEC", "hyperthermic intraperitoneal chemotherapy", "hot chemotherapy", "peritoneal malignancies", "peritoneal cancers", "peritoneal neoplasm", "peritoneal metastasis", and "outcomes". The Boolean search method was used in PubMed to combine the keywords and MeSH words. The full search strategy followed in PubMed is as follows: HIPEC OR Hyperthermic Intraperitoneal Chemotherapy OR Hot Chemotherapy OR (""Hyperthermic Intraperitoneal Chemotherapy"" [Mesh]) AND (""Hyperthermic Intraperitoneal Chemotherapy/adverse effects"" [Mesh] OR ""Hyperthermic Intraperitoneal Chemotherapy/methods"" [Mesh] OR ""Hyperthermic Intraperitoneal Chemotherapy/mortality"" [Mesh] OR ""Hyperthermic Intraperitoneal Chemotherapy/organization and administration"" [Mesh] OR ""Hyperthermic Intraperitoneal Chemotherapy/standards"" [Mesh] OR ""Hyperthermic Intraperitoneal Chemotherapy/therapeutic use"" [Mesh] ) AND Peritoneal Malignancies OR Peritoneal Cancers OR Peritoneal Neoplasm OR Peritoneal Metastasis OR (""Peritoneal Neoplasms/diagnosis"" [Mesh] OR ""Peritoneal Neoplasms/drug therapy"" [Mesh] OR ""Peritoneal Neoplasms/mortality"" [Mesh] OR ""Peritoneal Neoplasms/surgery"" [Mesh] OR ""Peritoneal Neoplasms/therapy"" [Mesh] ) AND outcomes. We applied filters for study type in PubMed, including articles categorized as a clinical trial, clinical trial phase I-IV, controlled trial, and randomized controlled trial. We also applied other filters such as studies involving humans only, studies in English language, and studies on cancer patients. In the Cochrane library, in addition to the year of the study mentioned above, we applied the filters including study type as trials and source as EMBASE. The searches were run twice by two authors (JLD and AK) before we retrieved the final set of articles, and we included any additional studies that were identified. All retrieved articles were scrutinized for relevance to our study and to exclude potentially irrelevant articles. 

### Study selection and data collection process

Study selection was carried out independently by two reviewers (JLD and AK). It was done in two phases. In phase A, after applying all the filters based on the inclusion and exclusion criteria, JLD and AK individually and independently screened the titles and abstracts of search results in all databases. Full-text articles were obtained for studies recognized as pertinent to this systematic review. In phase B, AK reviewed all full-text articles for eligibility and relevance. The articles that did not have information relevant to the objective of this study were excluded by AK. To avoid excluding pertinent articles, the articles excluded by AK were scrutinized again by JLD based on inclusion and exclusion criteria. JLD and AK thoroughly examined the full text of the studies that met the inclusion criteria independently to extract the data. Other authors independently checked and assessed the chosen articles for relevance. From the included study, the following data were extracted: number of patients in the HIPEC group and non-HIPEC group, number of patients with metastatic peritoneal disease in each group, survival rate, peritoneal recurrence-free survival (PRFS) rate, postoperative complications in each group. These data were entered in the Microsoft Excel sheet for analysis.

### Assessment of study risk of bias

Cochrane handbook for the systematic review of interventions was used to quality check the randomized controlled trials. Each domain was scored as low risk, high risk, or unclear accordingly for the chosen studies. JLD, AK, PT, and GK assessed all the selected randomized controlled trials. 

## Results

### Study selection outcome 

**Figure 2:**
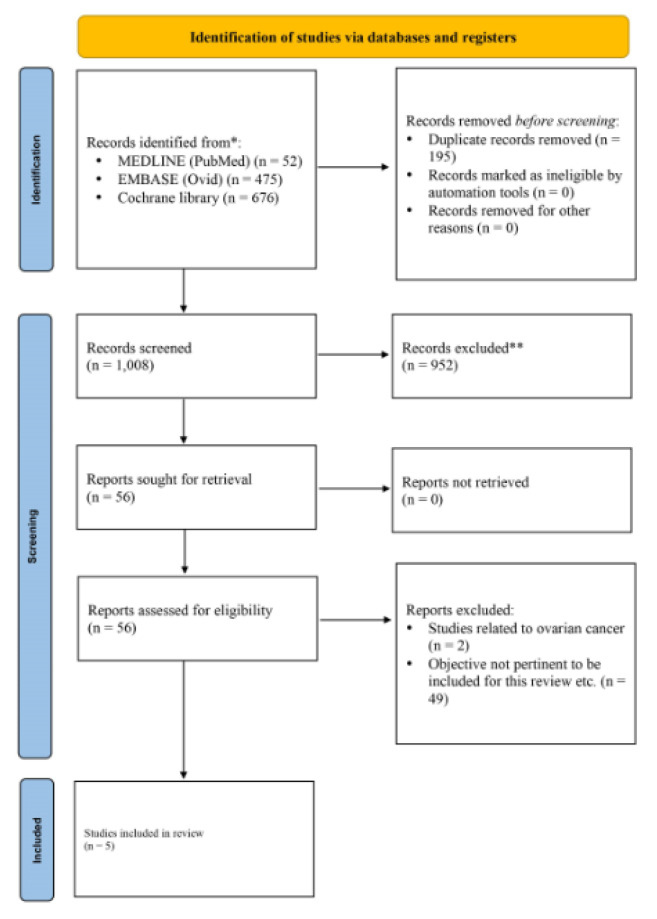
PRISMA Flow diagram

The initial database searches identified a total of 1,203 articles. Of these, 195 articles were found to be duplicates which were removed. We conducted the preliminary screening of these articles by reading the titles and abstracts and excluded 952 articles that were not relevant and did not meet the inclusion criteria. The full texts of the remaining 56 articles were reviewed for relevance and bias. After extensive evaluation of these 56 articles, 51 studies were excluded, thereby reaching a total number of five trials that were included for this systematic review. The PRISMA flow diagram is illustrated in Figure 2(Fig. 2).

### Study characteristics

Out of the five included studies, two studies were on patients with gastric cancer, and the other three studies were on patients with colorectal cancer. Three out of the five studies used adjuvant chemotherapy along with HIPEC. HIPEC was given to a total of 424 patients (116 gastric cancer patients and 308 colorectal cancer patients). The characteristics of the included studies are summarized in Table 1(Tab. 1) (References in Table 1: Beeharry et al., 2019[3]; Goéré et al., 2020[11]; Klaver et al., 2019[14]; Quenet et al., 2021[24]; Reutovich et al., 2019[25]). Of the included studies, Goéré et al.'s (2020[11]) PROPHYLOCHIP-PRODIGE 15 trial did not have the primary intention of assessing the role of HIPEC in the prevention of peritoneal recurrence in patients with colorectal cancer. However, on surgical exploration, a total of 34 patients without macroscopic peritoneal metastasis were found. Therefore, for these patients, the HIPEC would have played a role in preventing peritoneal metastasis, and for the remaining patients, it would have played a role in limiting the recurrence rates. Along with the patients who had macroscopic peritoneal metastasis in PROPHYLOCHIP-PRODIGE 15 trial, Quenet et al.'s (2021[24]) PRODIGE 7 trial was done on a total of 133 patients with peritoneal metastasis of colorectal origin received HIPEC for limiting the peritoneal recurrence. Of the included studies that studied the role of HIPEC in gastric cancer, the patients had locally advanced disease with tumor staging of T3 or T4. In two of the three studies on patients with colorectal cancer, the most common primary site of the tumor was the left colon, followed by the right colon.

### Peritoneal recurrence-free survival (PRFS) rates

In both the included studies on patients with gastric cancer, the PRFS was significantly higher in the group that received HIPEC as a prophylactic therapy to prevent recurrence. Both the studies reported 3-year PRFS. In Beeharry et al. (2019[3]) RCT, the three-year PRFS was 97 %. Out of the patients who underwent HIPEC, 38 patients had gastric cancer of stage pT4a. In Reutovich et al.'s (2019[25]) study, the three-year PRFS in the group that received HIPEC was 47 %, significantly higher than the control group. In this study, 63 patients had tumor stage pT4a, and 13 had tumor stage pT4b in the group that received HIPEC. In the same study, 71 patients had a tumor stage of pT4a, and seven patients had a tumor stage of pT4b in the control group. 

Of the studies on patients with colorectal cancer, the COLOPEC trial by Klaver et al. (2019[14]) studied a total of 100 patients with colorectal cancer who underwent HIPEC. Of these patients, 71 patients had stage pT4a, 16 patients had pT4b, and 10 had T2/T3 (perforation), and the remaining had T2/T3 (cT4). The 18-months PRFS was 80.9 %. However, the analysis showed it was not statistically significant. In the PROPHYLOCHIP-PRODIGE 15 trial, a total of 34 patients in the HIPEC group did not have peritoneal metastasis evidenced by surgical exploration at the time of initial presentation. For these patients, the HIPEC acted as a prophylactic therapy to prevent peritoneal recurrence. The other patients in the HIPEC group had evidence of peritoneal metastasis at the time of initial presentation (n = 37). Therefore, HIPEC acted as the therapy to reduce the recurrence in these patients. The three-year peritoneal recurrence-free survival was 59 % in the HIPEC group, and it was not statistically significant (Goéré et al., 2020[11]). In PRODIGE 7 trial, a total of 133 patients with metastatic colorectal cancer were given HIPEC to limit the recurrence. The three-year PRFS was 29.6 % in the HIPEC group, and it was not statistically significant (Quénet et al., 2021[24]).

### Postoperative complications and morbidity

Of the included studies on patients with gastric cancer, the data presented on postoperative complications varied among the studies. In the Reutovich et al. (2019[25]) study, the incidence of overall postoperative complications was 17.1 %. Two patients (2.6 %) had a severe esophageal anastomotic leak in the HIPEC group. Surgery-related complications such as abscess, pancreatitis, pancreatic fistula, abscesses were observed in 9 (12 %) patients in the HIPEC group. Other complications such as respiratory or cardiovascular complications were seen in 11 (14.5 %) patients. The incidence of complications in Beeharry et al. (2019[3]) RCT was 7.5 %, and there were no anastomotic leaks observed in any patients. 

In the included studies on patients with colorectal cancer, the postoperative complications were higher in the group that received HIPEC. Klaver et al. (2019[14]), in the COLOPEC trial, reported the incidence of postoperative complications to be 14 %. One patient had encapsulating peritoneal sclerosis. In the Goéré et al. (2020[11]) PROPHYLOCHIP-PRODIGE 15 trial, the incidence of postoperative complications was 41 %. Similar results were reported by Quenet et al. (2021[24]) in PRODIGE 7. In PRODIGE 7 trial, the rate of complications during days 1-30 of the postoperative period was 42 %. During days 1-30, 27 % intra-abdominal complications and 27 % extra-abdominal complications were observed in the HIPEC group. During days 31-60, the overall incidence of complications was 26 %. The patient characteristics, PRFS rate, and postoperative complications reported in the included trials are presented in Table 2(Tab. 2) (References in Table 2: Beeharry et al., 2019[3]; Goéré et al., 2020[11]; Klaver et al., 2019[14]; Quenet et al., 2021[24]; Reutovich et al., 2019[25]).

### Risk of bias

**Figure 3:**
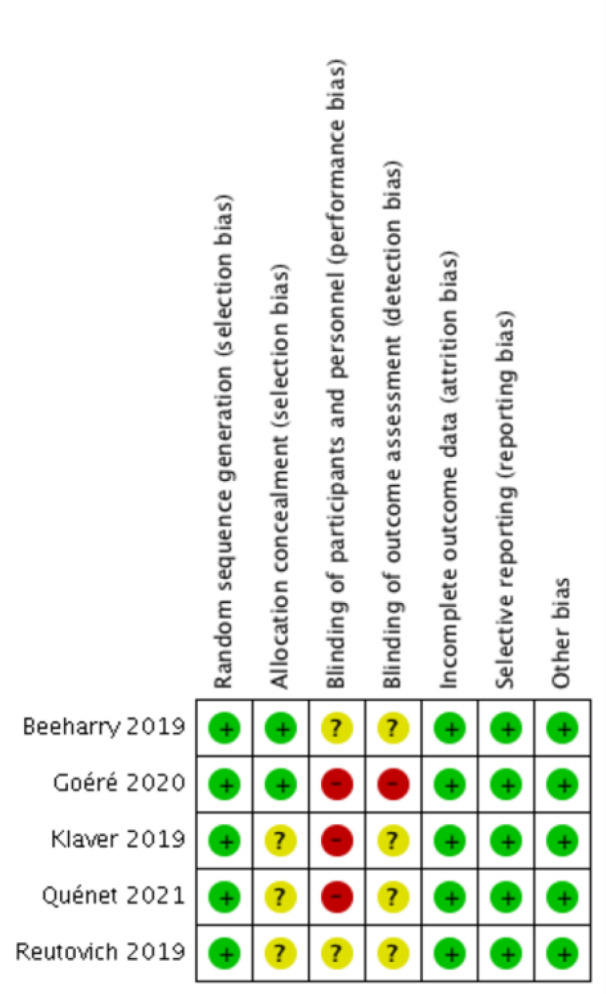
Risk of bias in all the included trials

**Figure 4:**
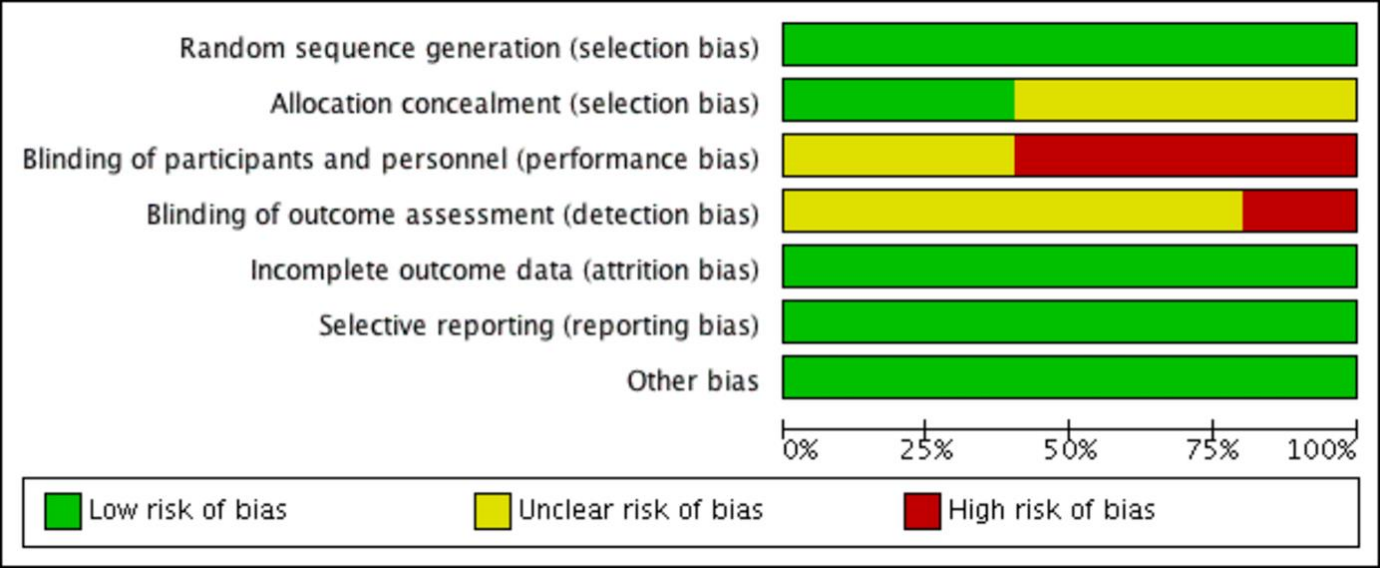
Summary of risk of bias assessment

Bias assessment was done using the Cochrane handbook for systematic reviews of intervention. Out of the five included studies, Beeharry et al. (2019[3]) trial used envelopes and chits for randomization. Other trials used computer-generated sequences. Allocation concealment was not reported explicitly in three studies. As all the studies involved patients who will undergo surgery for HIPEC, blinding of participants and personnel was not done in three studies, and it was unclear in the remaining three studies. Selective reporting and other biases were not observed in any of the included studies. The summary of the risk of bias in the included studies and the risk of bias in the individual studies are presented in Figure 3(Fig. 3) (References in Figure 3: Beeharry et al., 2019[3]; Goéré et al., 2020[11]; Klaver et al., 2019[14]; Quenet et al., 2021[24]; Reutovich et al., 2019[25]) and Figure 4(Fig. 4).

## Discussion

This systematic review of recently published randomized controlled trials was done to provide up-to-date information and the highest level of evidence regarding the role of HIPEC in the prevention and reduction of recurrence of peritoneal metastasis to the medical literature. Gastric cancer and colorectal cancer are the commonly diagnosed gastrointestinal malignancies worldwide. While HIPEC therapy is also given for other gastrointestinal malignancies like mucinous appendiceal cancer, we could not retrieve any recently published RCTs that evaluated the role of HIPEC in those patients. One of the most important reasons for poor prognosis in gastric cancer is recurrence (Allum and Fielding, 1990[1]; Sadeghi et al., 2000[26]). The common recurrence patterns of gastric cancer include peritoneal recurrence, distant metastasis, and regional recurrence, of which peritoneal recurrence is the most common type (Deng et al., 2011[6]). Thus preventing the recurrence of gastric cancer can possibly help in improving the survival of the patients. Compared to other treatment modalities for patients with gastric cancer, studies have reported that HIPEC has a positive outcome on improving overall survival (Yan et al., 2007[35]; Liang, 2016[16]; Coccolini et al., 2014[5]). However, studies in the literature lack in reporting the effectiveness of prophylactic HIPEC in preventing recurrence of peritoneal metastasis in patients with gastric cancer. Included trials in this review that studied the patients with gastric cancer had reported that the prophylactic HIPEC had an optimistic role in preventing disease recurrences as well as peritoneal recurrence with a significantly lower recurrence rate reported in all the included studies. 

All the included trials also reported that the overall survival rate was significantly higher in the patients who received HIPEC. Similar results were reported by Mi et al.'s meta-analysis of randomized controlled trials published in the year 2013[18]. The results of this meta-analysis showed that the peritoneal recurrence rate was significantly lower in the group that received HIPEC. This meta-analysis also reported that the overall survival was significantly higher in the group that received HIPEC. This demonstrates the fact that improving PRFS can directly improve the overall survival of patients with gastric cancer. On observing the limitation side of HIPEC, the incidence of postoperative complications did not differ significantly in the HIPEC group. Only Reutovich et al.'s study (2019[25]) reported two cases of fatal esophageal anastomotic leaks. In this study, the number of patients who underwent total, distal or proximal gastrectomies was not specified. Studies in the literature have reported that anastomotic leakage following total or subtotal gastrectomies would be relatively less in experienced centers (Piso et al., 2009[23]). Other included studies did not report any anastomotic leakage. Beeharry et al.'s study (2019[3]) reported a higher number of complications in the control group that received only cytoreductive surgery. Reutovich et al.'s (2019[25]) trial reported that the incidence of complications was not statistically significant in the HIPEC group. Beeharry et al. (2019[3]) also reported that the duration of hospital stay was significantly less in the HIPEC group. Postoperative complications prolong the hospital stay of the patients and contribute to morbidity and mortality. Liu et al.'s study (2016[17]) had reported grade 3-4 complications prolonged the duration of hospital stay and affected the patients quality of life that underwent total gastrectomy. With fewer postoperative complications and optimistic outcomes in improving peritoneal-free survival, prophylactic HIPEC appears to play a vital role in patients with gastric cancer, as evidenced by recently published randomized controlled trials. 

When it comes to colorectal cancer, prophylactic HIPEC therapy appears to be controversial, and data regarding the same are scarce in the medical literature. The first pilot study to determine the prophylactic role of HIPEC in colorectal cancer is by Virzì et al. (2013[33]). The authors studied twelve patients who were at high risk of developing peritoneal metastasis. The authors suggested that prophylactic HIPEC was safe and tolerable (Virzì et al., 2013[33]). Theoretically, as colorectal cancers invade and seed into the peritoneum, HIPEC could possibly prevent the implantation of the cancer cells into the peritoneum by killing them (Perez et al., 2021[22]). Sammartino et al. (2012[27]) reported 4 % peritoneal recurrence in their study. However, the authors could not conclude whether the reduced peritoneal recurrence rate was solely due to HIPEC. Various studies in the literature that were not RCTs reported low peritoneal recurrence rates and improved PRFS when prophylactic HIPEC was used to prevent peritoneal metastasis in patients with colorectal cancer (Sammartino et al., 2012[27]; Tentes et al., 2011[31]). 

All the recently published RCTs have reported contradicting results on the prophylactic and therapeutic use of HIPEC to prevent and limit peritoneal recurrence in patients with colorectal cancer. All the trials included in this review reported that there was no significant improvement in the PRFS in patients who received HIPEC. In the COLOPEC trial, the 18-month PRFS in the HIPEC group was 80.9 %, whereas, in the control group, it was 76.2 %. The HIPEC group received both adjuvant chemotherapy and HIPEC. The control group received only adjuvant chemotherapy. One of the limitations reported by the authors was the administration of adjuvant chemotherapy later in the experimental group than in the control group though all patients received adjuvant chemotherapy within the recommended period (Klaver et al., 2019[14]). The PROPHYLOCHIP trial, which reported a 3-year PRFS rate, did not show a significant difference between the HIPEC group (61 %) and the control group, which had only surveillance (59 %) (Goéré et al., 2020[11]).

In PRODIGE 7 trial, the PRFS did not show any significant difference between the HIPEC and the non-HIPEC group, which received cytoreductive surgery along with systemic chemotherapy (Quénet et al., 2021[24]). In all three trials, HIPEC was given for 30 minutes duration. This leads us to investigate the effectiveness of the shorter duration HIPEC. In the COLOPEC trial, Klaver et al. (2019[14]) suggested that a shorter duration HIPEC for 30 minutes may not lead to significant exposure of the tumor cells to the chemotherapeutic to attain an effective anti-tumor response. Quenet et al. (2021[24]) also suggested the same. The efficacy of the shorter duration HIPEC can only be clarified by ongoing and future RCTs. All the trials included also reported that HIPEC did not show significant improvement in the overall survival rate.

In addition, the complications were higher among the patients who received HIPEC. In the COLOPEC trial, postoperative complications occurred in 12 patients who received HIPEC. Two patients had an anastomotic leak. One patient who received HIPEC developed encapsulating peritoneal sclerosis that was subsequently treated (Klaver et al., 2019[14]). Averbach et al.'s study (1996[2]) reported a higher incidence of anastomotic leak in the patients who received HIPEC when extensive colon resection was done. The included trials reported frequent postoperative complications in the HIPEC group. The complications further affect the survival negatively, thereby leading to further reduction in the survival rate. Thus, along with decreased efficiency to prevent and control peritoneal metastasis, HIPEC treatment and prophylaxis seem to negatively impact patients with colorectal cancer. As this review included only three trials, the ongoing and future trials may show a clarifying and explicit result on the outcomes of a patient with colorectal cancer undergoing HIPEC treatment for prevention and control of peritoneal metastasis.

One of the most important limitations of this review is the number of studies and the type of studies chosen. Our primary objective was to provide the highest level of evidence of updated information in the field of surgical oncology and HIPEC therapy. Considering the level of biases in other types of studies and the level of evidence available, only RCTs were chosen. HIPEC therapy is not available worldwide, and even in the available places, only a few experienced and trained experts are available to do the procedure. The number of RCTs available in the literature involving HIPEC is very scarce. Most of the RCTs get called off due to a considerable loss of patient follow-up and leading to deficiency in the data. Considering the fact of the presence of other systematic reviews and meta-analyses of previously published RCTs, we chose to systematically analyze only the recently published RCTs to provide up-to-date information, which may serve as the highest level of evidence to the medical literature.

In conclusion, HIPEC appears to be effective in preventing peritoneal metastasis in patients with locally advanced gastric cancer with minimal postoperative complications. However, in patients with advanced colorectal malignancy, HIPEC does not seem to play a crucial role in preventing and controlling peritoneal metastasis. Moreover, the risk of postoperative complications is higher and thus further contributing to morbidity and mortality. The ongoing and future RCTs need to prove the prophylactic effectiveness of HIPEC in patients with colorectal cancer.

## Declaration of interest

None.

## Funding

This research did not receive any specific grant from funding agencies in the public, commercial, or not-for-profit sectors.

## Figures and Tables

**Table 1:**
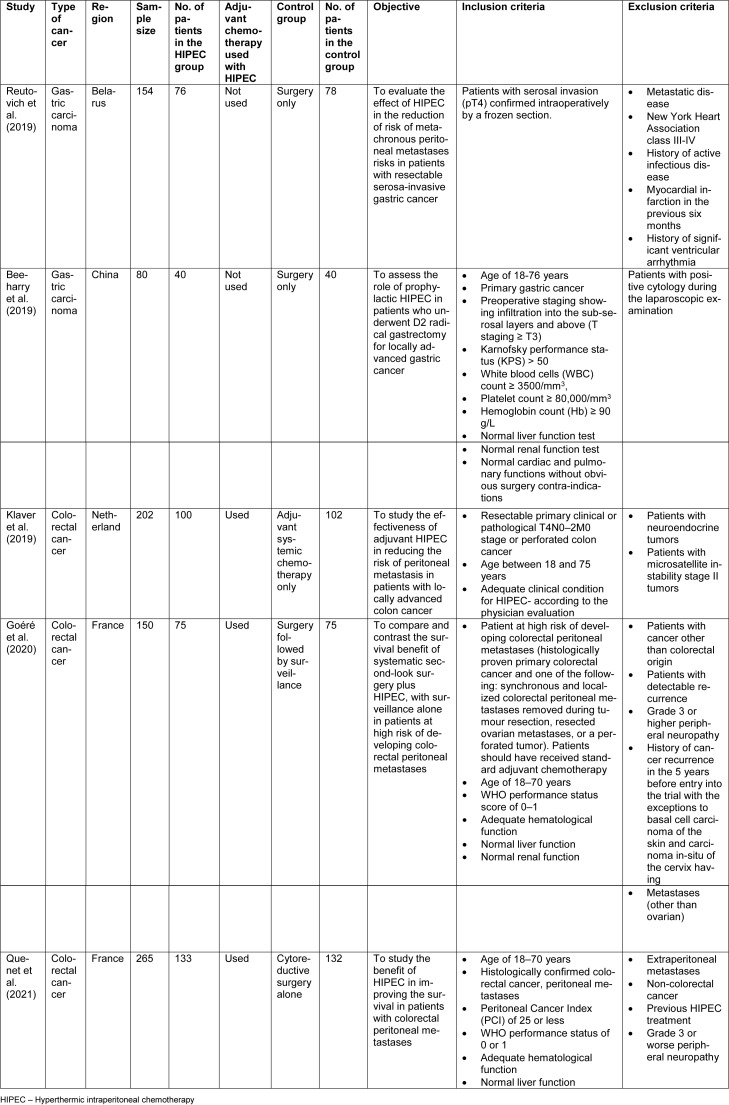
Characteristics of the included randomized controlled trials (RCTs)

**Table 2:**
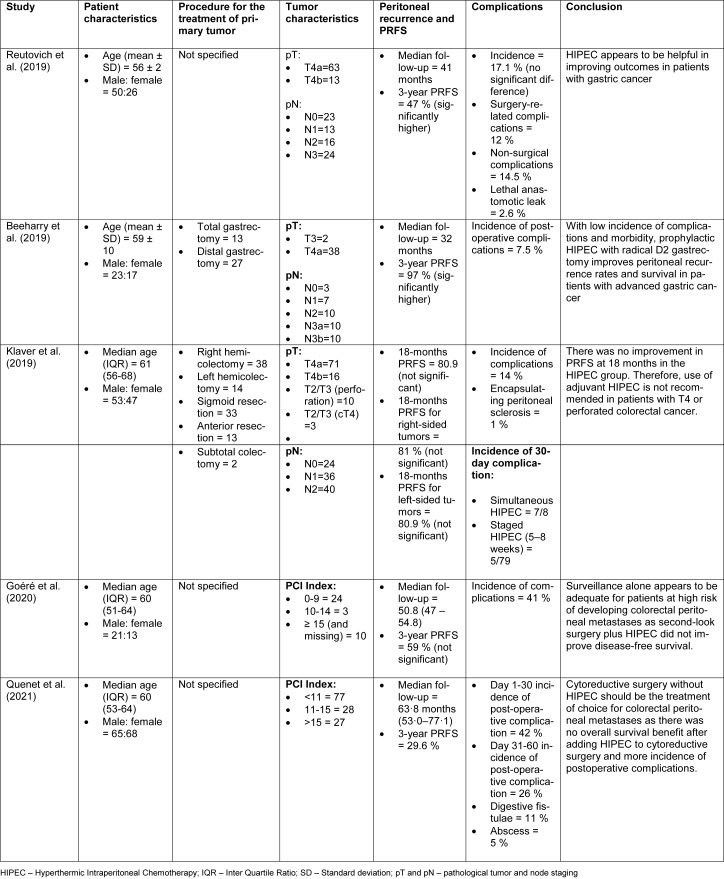
Patient characteristics, peritoneal recurrence-free survival, complications reported in the included studies
